# Individualized reconstruction for severe periprosthetic fractures around the tumor prosthesis of knee under assistance of 3D printing technology: A case report

**DOI:** 10.1097/MD.0000000000012726

**Published:** 2018-10-19

**Authors:** Qing Han, Xue Zhao, Chenyu Wang, Bingpeng Chen, Xiaonan Wang, Ziyan Zhang, Kesong Zhang, Yuhao Zheng, Jincheng Wang

**Affiliations:** a Orthopedics Center, The Second Hospital of Jilin University; b Department of Endocrinology and Metabolism, The First Hospital of Jilin University, Changchun, China.

**Keywords:** 3-dimensional printing, individualized tumor prosthesis of knee, periprosthetic fractures, preoperative design, tumor prosthesis

## Abstract

**Introduction:**

Periprosthetic femoral fractures (PFF) around tumor prosthesis of knee are stubborn problems for surgeons, huge bone defect and inappropriate biomechanics of the revision implant design can be disaster for reconstruction. With the development of three-dimensional (3D) printing technology, surgeons participate more in precise preoperative design and simulation for treatment of such fractures. In this study we explored an accurate and feasible way to restore normal anatomy and function of the knee joint with 3D printing technology.

**Case presentation:**

**Rationale:** This report explored an accurate and feasible way to treat PFF around tumor knee prosthesis in a 32 years old women with 3D printing technology, which restored normal anatomy and function of the knee joint. **Patient concerns:** Pain in left thigh lasted for 10 months after resection of left femoral chondroma and knee joint replacement four years ago. **Diagnoses:** periprosthetic femoral fractures (PFF) around tumor knee prosthesis.

**Interventions:**

CT images of the patient were collected and reconstructed. Parameters of bilateral femurs were virtually sliced and measured. Novel femoral stem and nail paths were specially designed by doctors according to these parameters. The prosthetic femoral stem components and navigator were customized by engineers according to the doctor's design. The residual femoral resin model, customized components and navigator were printed with Stereo Lithography Apparatus 3D printer. The shape-preconcerted allograft bone was selected as patch for the bone defect before operation with the printed bone model. All the steps were simulated preoperatively with the models printed, and then the operation was carried out. **Outcomes:** The operation was successfully performed. The postoperative x-ray image, MSTS93 scores were examined and the function restoration sustained well in the follow-up period from 1 month to 27 months. **Lessons:** 3D printing and medical interaction are key points in complex PFF cases.

**Conclusion:**

As for PFF of the complex tumor of knee, preoperative design and simulation with 3D printing technology may provide more accurate and effective operative outcome than traditional methods, which might be considered as a method suitable for popularization in complex and severe cases.

## 1 Introduction

For patients with proximal tibia or distal femoral tumors, complications such as infection, aseptic loosening, and periprosthetic femoral fractures (PFFs) often happened after conventional knee tumor prosthesis replacement. The patient with periprosthetic fractures in femur is usually involved with large-scale bone defects, inadequate bearing bone, and accompanied peripheral soft tissue damage. Most of these cases need a second revision surgery, which is full of risk and challenge. It is reported that the long-term survival rate of similar cases is comparatively low, the 5-year survival rate is 57% to 93%, whereas 10-year survival rate is 60% to 88%.^[1,2]^ Some of the reasons can be attributed to the prosthesis design and the operation. Therefore, the precise preoperative design and simulation by surgeon is a reliable and hopeful way to successful operation and fine follow-ups. Now the development of 3-dimensional (3D) printing technology brought such possibilities for doctors and this technology enormously enhance the medical-engineering interaction.^[3]^ 3D printing technology is widely applied in orthopedics in recent years. From preoperative design to operative simulation, from individual customization of operative guide plate to the customization of the individual implant, all of these applications have been reported to enhance the accuracy and individuation of orthopedic surgeries.^[4–9]^ The biggest advantage of this technology is to provide a platform for both doctors and engineers to communicate thoroughly as well as to represent the types and relations of fracture positions accurately.^[10,11]^ In this study, we have explored a novel method for design of the femur part in a patient with complex knee tumor PFF, which has not been reported by others. In addition, we have taken full advantages of 3D printing technology to design the prosthetic components and navigator. Through this process, we want to explore an individual and effective way of preoperative design and simulation with this 3D printing technology in such complex cases.

## 2 Case presentation

### 2.1 Ethical statement

All procedures performed in studies involving human participants were in accordance with the ethical standards of the ethical committee in our hospital and with the Helsinki declaration and its later amendments or comparable ethical standards. Informed written consent was obtained from the patient for publication of this case report and accompanying images. This study was performed in accordance with relevant guidelines and regulations.

### 2.2 Clinical data

**Figure 1:**
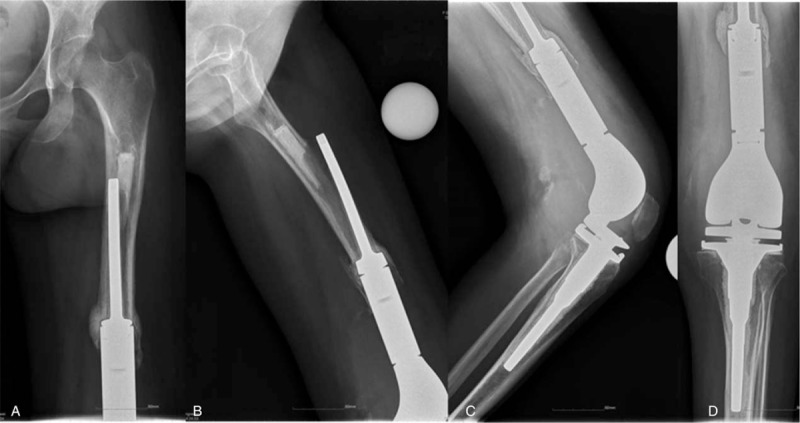
Patient's anteroposterior and lateral x-ray image. A, Anterior-posterior x-ray of femur. B, Medial-lateral x-ray of femur. C, Anterior-posterior x-ray of knee. D, Medial-lateral x-ray of knee.

A 32-year-old female patient was diagnosed as “left femoral chondroma” because of pain in the left thigh 4 years ago and received the left femoral tumor resection operation. The pain was not relieved after operation, so enlarged tumor resection and total knee arthroplasty were performed 1 year later. The postoperative biopsy revealed that it is still the left femoral chondroma. The patient recovered well after operation. The pain recurred in the left thigh 10 months ago without obvious causes, aggravated while moving and eased after rest. The x-ray image showed that the femoral prosthesis loosening after left knee arthroplasty. The patient came to the hospital with a wheelchair. The left lower limb was swelling with extorsion deformity. The left thigh and knee joint were significantly swelling compared with the uninjured side. A postoperative scar with 25 cm in length was seen in front of left knee joint. There were no obvious skin damage and subdermal ecchymosis in the incision. The tenderness was obvious in the middle segment of left thigh and an irregular mass could be touched with hard nature. The range of motion (ROM) was limited. The patient was not co-operative with physical examination due to the pain in the left knee. The flexion and extension were significantly restricted but the ROM was in normal range in the hip joints. The ROM of right hip and knee joints was normal. The bilateral dorsalis pedis arterial pulses were good. The dorsiflexion of ankle and each toe was good. The pathological reflex was not found. The blood routine and erythrocyte sedimentation rate had no signs of infection. The patient had long-term laminated object manufacturing. Arterial venous color Doppler ultrasound for bilateral limbs showed that no significant abnormity was found in arteries and veins of both limbs (Fig. 1).

### 2.3 3D reconstruction and prosthetic component design

**Figure 2:**
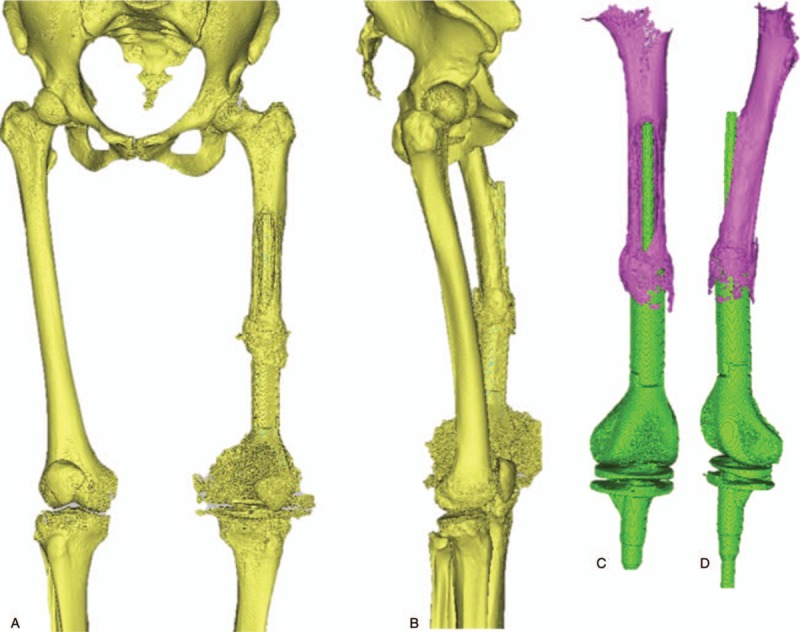
Three-dimensional (3D) reconstruction of relations between prosthesis and fracture position. A and B, The whole reconstruction of the femur and prosthesis. C and D, The detail of the periprosthetic fracture.

**Figure 3:**
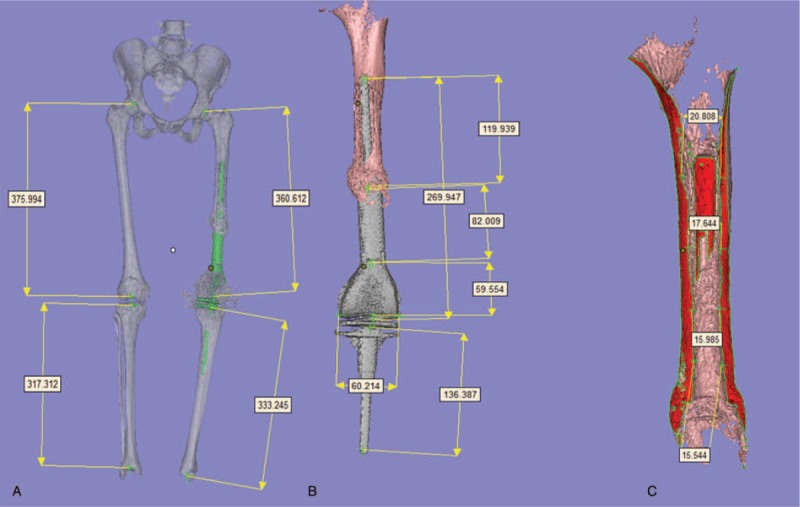
Parameters of prosthesis measured at each part. A, The whole femur and tibia length. B, Each part of the prosthesis. C, Medullary cavity of the residual femur.

The CT data of both lower limbs were imported into the Materialise Mimics 17.0 software (Materialise, Leuven, Belgium) for 3D reconstruction of bilateral femur, tibia, fibula and also the modular prosthesis. The length of bilateral femurs and size of medullary cavity at each height were measured after reconstruction. The length of affected fracture position, length of residual femur, position of proximal vertex of femoral prosthesis, and length of each part of modular prosthesis were measured. Meanwhile, the femur was split apart in Materialise Magics RP 18.0 software (Materialise) to measure the thickness of affected femoral marrow cavity at each position. The bone cement was distinguished at the proximal prosthesis and the starting and ending points of bone cement were recorded so as to clean them up during operation. The superior segment of detachable femoral prosthesis was measured about 119.9 and 8 mm from the top of bone cement to the inferior lesser trochanter, 25 mm of vertical distance to the vertex of lesser trochanter. The length of the bone cement was 65 mm. The available sclerotin was approximately 140 mm from the vertex of lesser trochanter to the lower end. The thinnest residual medullary cavity was approximately 15.544 mm. The straight-line distance was approximately 95 mm from proximally extruded part of prosthesis to the cupular part. The length of complete residual bone ring was approximately 31.5 mm. The retainable residual bone was 105 mm in length at fracture position. The femoral isthmus of normal side was 13 mm and the isthmus to the inferior less trochanter was 10 mm. Distance from the isthmus to the midpoint of distal end of femoral condyle was 200 mm (Figs. 2 and 3).

**Figure 4:**
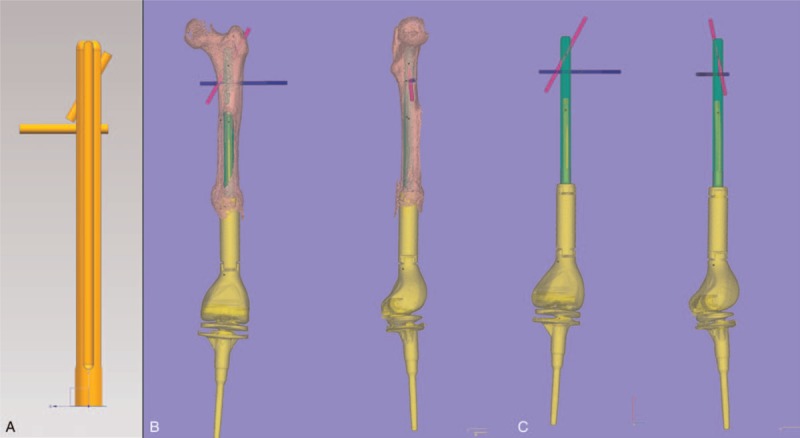
Designs for prosthetic components and nail paths. A, The redesigned stem. B, Prosthesis and femur assembling test. C, Final effect of the whole prosthesis design.

Because other parts of the tumor knee modular prosthesis were rigidly fixed and no infection was found in the patient, the guideline of our design is to replace the component part in fracture position, which could not support the bone any longer. According to the data measured above, the residual bone was imported into UG NX 10.0 (Siemens PLM Software). A femoral prosthetic stem with 14 mm in diameter and 190 mm in length was designed. The top of stem was 1 cm away from the pyriform sinus. The central part was designed at 20 and 28 mm with a 6 mm of nail hole, which presented 90° and 30° angles, respectively to the prosthesis stem to make sure the stability of the stem. According to the position of the nail hole and screw position of fixed components, a navigator was designed for blinding lock and intraoperative installation. The customization for prosthetic components was carried out after the final draft of designed 3D diagram was made. Meanwhile, allograft bones were selected based on the measured size of defect cortex bone to guarantee the optimal match between bone lamella and defect (Fig. 4).

### 2.4 3D printed model and preoperative simulation

**Figure 5:**
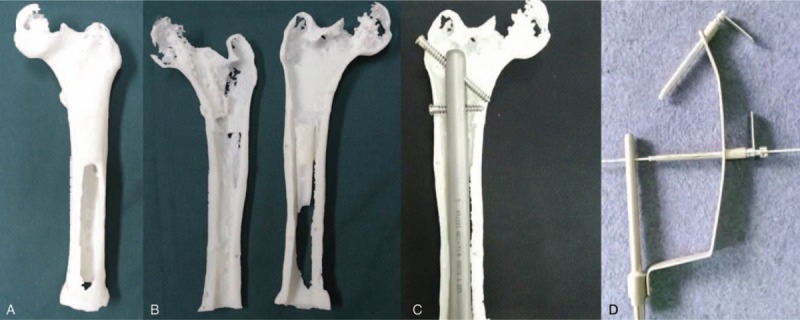
Three-dimensional (3D) printed model matched with customized prosthesis and navigator. A, The residual femur. B, The residual femur in halves. C, Assembling test. D, Navigator design.

The femoral model at the affected side in this patient was separated from the prosthesis in mimics. Standard Template Library (STL) file was exported after removing the noise and imported into Magics RP 18.0 for triangle repairing. The angle was adjusted for a longitudinal split in an optimal angle. Two pieces of spilt sclerite were lettered, labeled, and repaired respectively. An upright column and groove at a suitable position were as well as added thereby to make them be spliced and installed completely. Then these models were printed and reprocessed after the print process. The model was matched with the well-customized prosthesis and the prosthesis could be inserted into the model successfully. The position of nail path was confirmed with navigator (Fig. 5).

### 2.5 Operative procedures

**Figure 6:**
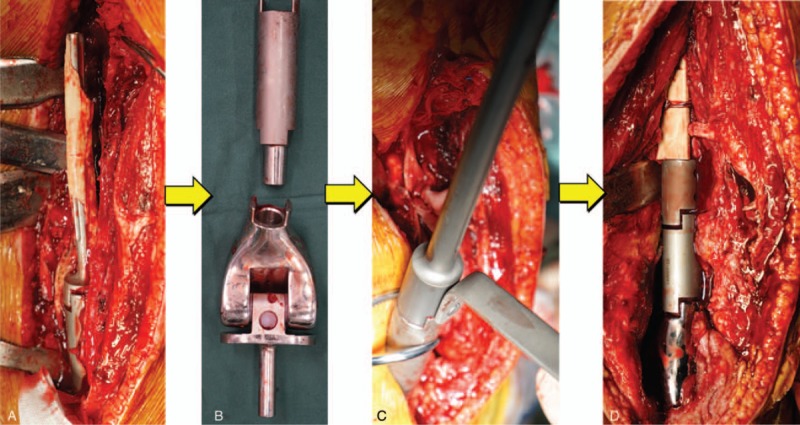
Customization process of femoral prosthesis after prosthesis taken out. A and B, Removal of the prosthesis. C, New part assembled successfully. D, Implantation of the prosthesis.

**Figure 7:**
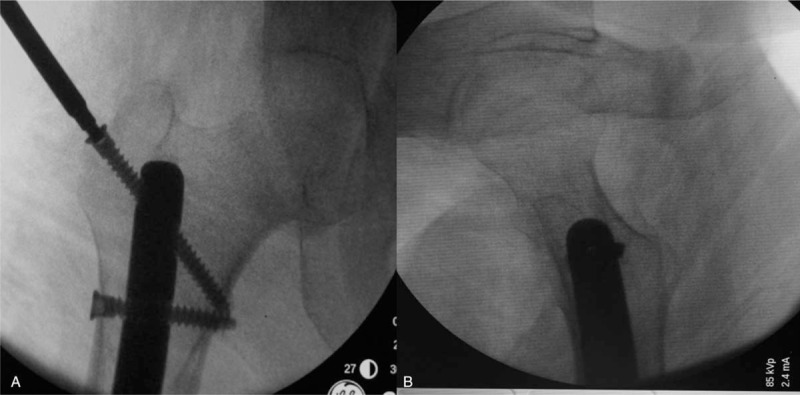
Successful nailing by navigator under intraoperative x-ray. A, Anterior-posterior x-ray of femur during operation. B, Medial-lateral x-ray of femur during operation.

A 35 mm incision was taken at lateral femur starting from the small tuberositas and down to the lateral knee joint. The skin, subcutaneous tissue, and fascia lata were cut open one by one. The lateral femoral muscle fibers were bluntly dissected to pull open toward front and back to expose the femur. The prosthesis stem at femur side passed through the femur toward the front. Plenty of bloody fluid was discharged. The samples were taken for bacterial culture and pathological specimens. The prosthesis stem was taken down, the scars, and proliferous bony fibrous tissue were cleaned up. The customized intramedullary fixation device was installed from the femoral side. Two locking nails were implanted into the proximal femur under guide of navigator, then they were successfully matched and confirmed by intraoperative x-ray. The allogeneic cortical bone plate selected before operation was fastened to the bone defect of anterior femur by 3 wires. After surely fastening, the bone cement containing antibiotics was used to cover the surface of the prosthesis. The wound (Figs. 6 and 7) was conventionally washed and sutured after cement solidification.

### 2.6 Pathological examination results for intraoperative taken tissues

**Figure 8:**
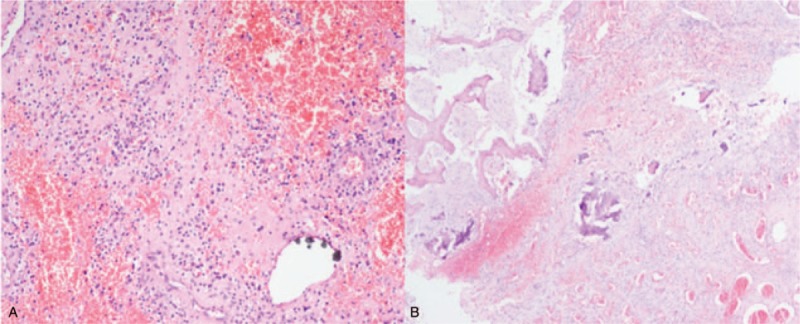
Tissue slice taken out around the fracture. A, Hyperplastic fiber tissue. B, Bone tissue and calcification.

**Figure 9:**
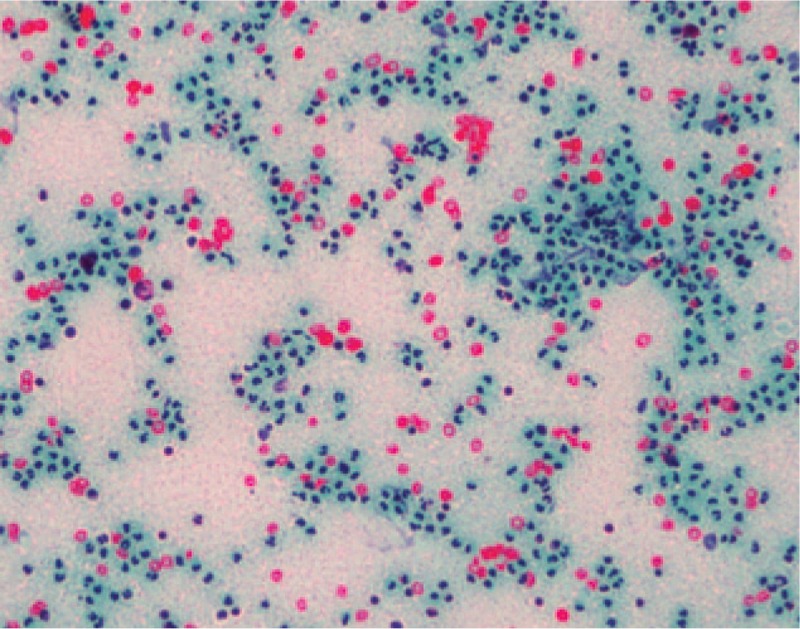
Intraoperatively submitted periprosthetic interstitial fluid.

Massive acute and chronic inflammatory cell infiltration was seen in the peripheral hyperplastic fibrous tissue of the prosthesis, with bleeding, necrosis, hemosiderosis, and a little bone necrosis. There were rich blood vessels and granulation tissue formation in parts of prosthesis. Bone tissue and calcification (Fig. 8) were seen in another piece of prosthesis’ lateral hyperplastic tissue. More neutrophils, phagocytes, and a small amount of lymphocytes were seen in the submitted interstitial fluid at periprosthetic fracture position, but no malignant cells were found (Fig. 9).

### 2.7 Outcomes and follow-up

**Figure 10:**
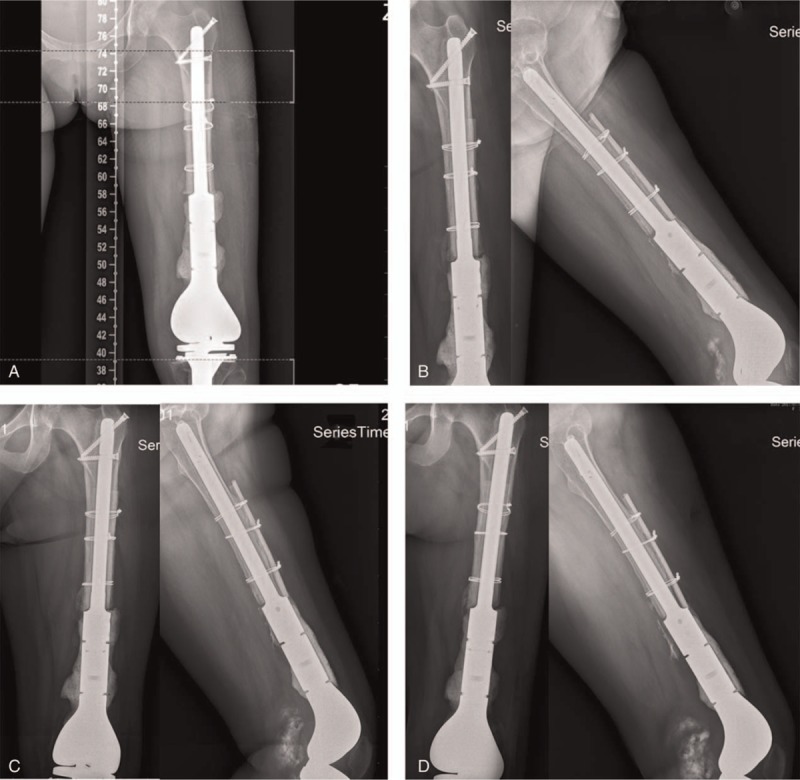
Postoperative x-ray images. A, Postoperative x-ray image. B, 6-Month follow-up. C, 16-Month follow-up. D, 22-Month follow-up.

**Figure 11:**
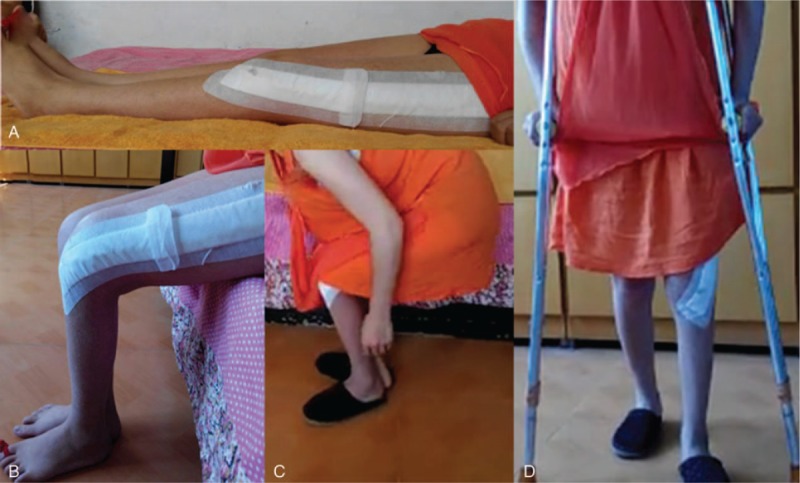
Follow-up after 1 month. A, Comparison in length in both lower limbs. B, Bending. C, Independent squatting. D, Walking with double crutches.

**Figure 12:**
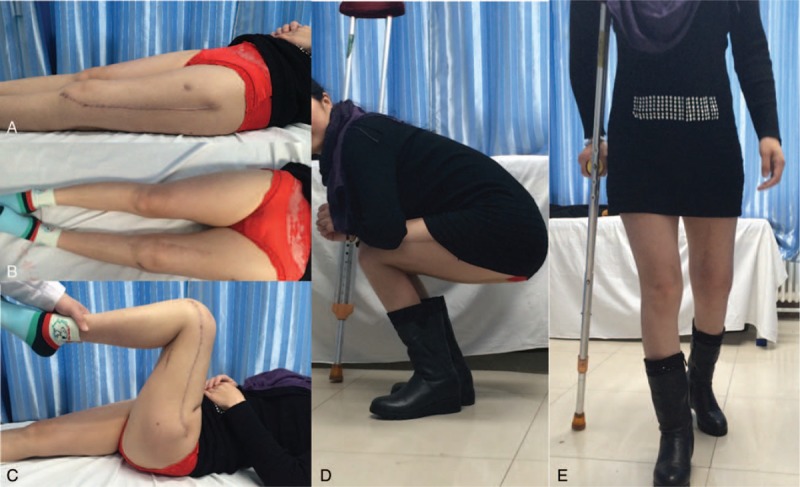
Follow-up after 6 months. A, Extension of the knee. B, Comparison of bilateral legs. C, Flexion of the knee. D, Independent squatting. E, Walking with single crutches.

**Figure 13:**
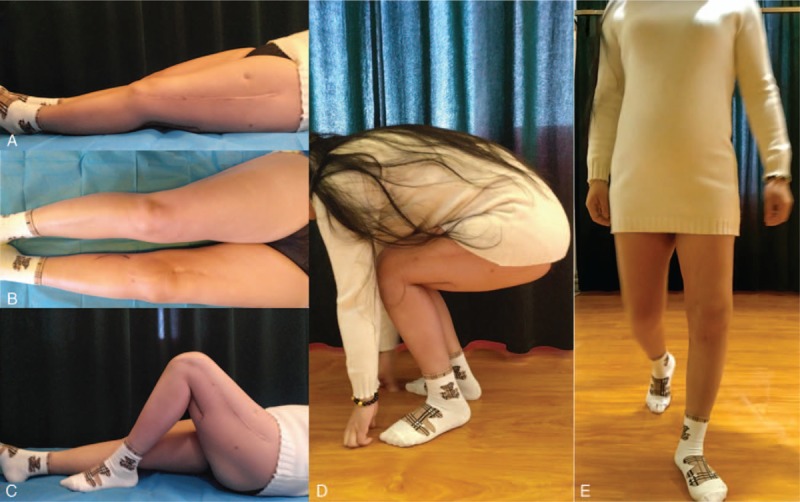
Follow-up after 27 months. A, Extension of the knee. B, Comparison of bilateral legs. C, Flexion of the knee. D, Independent squatting. E, Independent walking.

The counterpoint and alignment were well shown in postoperative anteroposterior film of x-ray postoperatively, 6, 16, and 22 months (Fig. 10). The length of both lower limbs was almost equal. The squat can be independently conducted. The angle of knee joint bend was about 80° while squatting. The patient can walk by herself with the help of crutches within 1 month postoperatively and walk by herself for limited distance after 12 months (Figs. 11–13). Flexion and extension of her knee joint restored to an acceptable range, necessary daily movement such as squatting and standing can be done by herself. Her body weight gain from 48 kg, 55 to 63 kg at 1, 6, and 27 months of postoperative follow-up. The patient's MSTS93 score was improved from 0 point before operation to 14 point at 1 month, 18 point at 6 months, 23 point at 9 months, 26 point at 16-month follow-up, and 28 point at 27-month follow-up.

## 3 Discussion

The cases of patients with tumor knee PFF have been rarely reported, so does the causes of these cases. Common causes include tumor recurrence, infection, aseptic loosening, periprosthetic fractures, broken prosthetic stem, hinge structure failure, etc.^[12,13]^ It is reported that femoral anterior arch may lead to supracondylar fracture of femur. Approximately 10% to 46% of fractures have recurred at femoral anterior arch. And 3 mm anterior defect will lead to 30% of strength decreases of antitorsional bone. In this case, no infection was found in preoperative and intraoperative examinations. The prosthesis extruded out from anterior femur. The possible causes of PFF may also be the biomechanical causes such as mismatch of femur arc. The stress caused by mismatch between angle of femoral anterior arch in tumor prosthesis’ femoral part and angle of femur arch was concentrated on certain point of the bone, resulting in femoral cortex being slowly damaged. Compared with periprosthetic fractures of regular total hip arthroplasty, cases involved in extensive resection of distal femoral is highly challenged in treatment. As for revision related to infection, prosthesis is needed to be removed due to infection in stage II revision surgery after anti-infective therapy, other revisions can be completed in stage I. Moreover, according to intraoperative pathological examinations, if the conditions permit, the parameters of metal ionized water level in serum shall be tested to study whether the rotated hinge knee-induced residues have caused the body responses.^[14]^ According to the detailed and comprehensive information of this case, we decided to change only the extruded part of the tumor knee joint. So an accurate design in line with the specific situation of this patient is the key to success of this revision. The prosthesis should be carried out by the doctor and the engineer together to make it secure.

Traditional method of customizing prosthesis is engineer's job, who will make the design according to the 2D x-ray image. And there was no close interaction between doctors and engineers. As a usual result, the quality of restored force line and anatomy might not maintain for a long period, and thus demand of such a young patient cannot be satisfied. Once several lantern rings are applied to make up for length, the total strength of the force line would be changed. So the nail hole and screw position must be confirmed by the surgeon and then be performed by the engineer. In addition, surgeons usually get the prosthesis without a clear understanding of the case. However, in this case, surgeons can manipulate the design and customization in the computer screen. The customization is carried out after the success of matching simulated operation. The whole operation process and osteotomy scope can be confirmed before operation either in the screen or on the 3D printed models. As for similar cases, a whole femoral prosthesis customization and replacement have been performed in similar cases abroad so as to ensure the patients have enough strength supporting^[15]^ at the affected femur. However, patient in this case was young, the proximal femur was retained intactly and structure of the tumor prosthesis was modular, so we considered to replace part of this prosthesis at affected femoral side and meanwhile repair the bone defect with allograft at fracture position according to preoperative design.^[16–18]^ Postoperative x-ray showed that the counterpoint and alignment were good at affected lower limb at 22 months follow-ups. The patient even could squat in a satisfied angle with aid at only 1 month postoperative follow-up and could still perform this movement by herself at 27th month, which can be an evidence of the short-term effect of this revision method.

Because only 1 patient was included in this study and the focus of this article was put in the process of medical engineering and preoperative design and simulation, more researches should be done to compare this method with traditional ones and follow-ups with long time should also be provided.

## 4 Conclusion

For the patients with complex PFF of tumor knee prosthesis, the preoperative design and simulation with 3D printing technology can enhance the interaction between doctors and engineer, which would maximally promote the possibility of operation success. This method can also improve the feasibility of the prosthesis design and surgery process, which guaranteed a precise and reliable operation outcome and follow-up.

## Author contributions

**Conceptualization:** Jincheng Wang.

**Data curation:** Chenyu Wang, Xiaonan Wang.

**Formal analysis:** Ziyan Zhang.

**Funding acquisition:** Jincheng Wang.

**Methodology:** Qing Han, Kesong Zhang.

**Resources:** Yuhao Zheng.

**Software:** Kesong Zhang.

**Supervision:** Bingpeng Chen, Jincheng Wang.

**Writing – original draft:** Qing Han, Xue Zhao.

**Writing – review and editing:** Bingpeng Chen, Jincheng Wang.
